# Observed coupling between air mass history, secondary growth of nucleation mode particles and aerosol pollution levels in Beijing†

**DOI:** 10.1039/d1ea00089f

**Published:** 2022-01-19

**Authors:** S. Hakala, V. Vakkari, F. Bianchi, L. Dada, C. Deng, K. R. Dällenbach, Y. Fu, J. Jiang, J. Kangasluoma, J. Kujansuu, Y. Liu, T. Petäjä, L. Wang, C. Yan, M. Kulmala, P. Paasonen

**Affiliations:** a Aerosol and Haze Laboratory, Beijing Advanced Innovation Center for Soft Matter Science and Engineering, Beijing University of Chemical Technology Beijing China; b Institute for Atmospheric and Earth System Research/Physics, Faculty of Science, University of Helsinki Helsinki Finland simo.hakala@helsinki.fi; c Finnish Meteorological Institute Erik Palmenin Aukio 1 Helsinki Finland; d Atmospheric Chemistry Research Group, Chemical Resource Beneficiation, North-West University Potchefstroom South Africa; e Extreme Environments Research Laboratory, Ecole Polytechnique Fédérale de Lausanne (EPFL) Valais Sion 1951 Switzerland; f Laboratory of Atmospheric Chemistry, Paul Scherrer Institute 5232 Villigen Switzerland; g State Key Joint Laboratory of Environment Simulation and Pollution Control, School of Environment, Tsinghua University Beijing China; h Joint International Research Laboratory of Atmospheric and Earth System Sciences, Nanjing University Nanjing China; i State Key Laboratory of Atmospheric Boundary Layer Physics and Atmospheric Chemistry (LAPC), Institute of Atmospheric Physics, Chinese Academy of Sciences Beijing 100029 China

## Abstract

Atmospheric aerosols have significant effects on the climate and on human health. New particle formation (NPF) is globally an important source of aerosols but its relevance especially towards aerosol mass loadings in highly polluted regions is still controversial. In addition, uncertainties remain regarding the processes leading to severe pollution episodes, concerning *e.g.* the role of atmospheric transport. In this study, we utilize air mass history analysis in combination with different fields related to the intensity of anthropogenic emissions in order to calculate air mass exposure to anthropogenic emissions (AME) prior to their arrival at Beijing, China. The AME is used as a semi-quantitative metric for describing the effect of air mass history on the potential for aerosol formation. We show that NPF events occur in clean air masses, described by low AME. However, increasing AME seems to be required for substantial growth of nucleation mode (diameter < 30 nm) particles, originating either from NPF or direct emissions, into larger mass-relevant sizes. This finding assists in establishing and understanding the connection between small nucleation mode particles, secondary aerosol formation and the development of pollution episodes. We further use the AME, in combination with basic meteorological variables, for developing a simple and easy-to-apply regression model to predict aerosol volume and mass concentrations. Since the model directly only accounts for changes in meteorological conditions, it can also be used to estimate the influence of emission changes on pollution levels. We apply the developed model to briefly investigate the effects of the COVID-19 lockdown on PM_2.5_ concentrations in Beijing. While no clear influence directly attributable to the lockdown measures is found, the results are in line with other studies utilizing more widely applied approaches.

## Abstract 2

Growth of nucleation mode particles, modulated by air mass exposure to anthropogenic emissions, plays an important role in pollution development.

## Graphical abstract

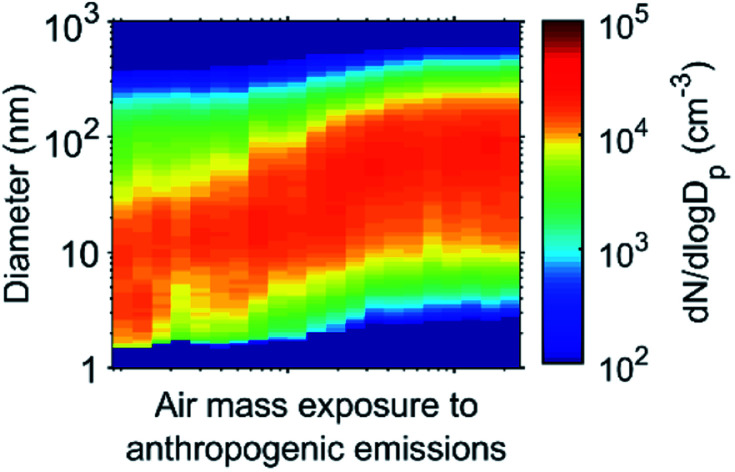

**Environmental significance**

Understanding the causes and formation mechanisms of urban air pollution are vital for its efficient mitigation. While the significant role of secondary aerosol formation to PM_2.5_ pollution in Beijing is commonly known, this study assists in understanding the sources of the seed particles for the pollution. We show that PM_2.5_ pollution in Beijing has its roots all the way in the smallest nucleation mode particles produced by new particle formation events and direct emission of small particles from *e.g.* traffic. Therefore, focusing on controlling these sources, and the sources contributing to secondary growth of aerosol, could provide efficient mitigation solutions. We also clearly display the crucial need to account for air mass history when investigating aerosol formation in Beijing.

## 1 Introduction

Despite ongoing pollution control measures, serious haze episodes with adverse health effects remain problematic in Beijing, China.^1–3^ The haze episodes are periods with elevated concentrations of aerosol particles in the accumulation mode size range (particle diameter 100 nm–1 µm), which are large enough to affect visibility by scattering and absorbing solar radiation. Accumulation mode particle concentrations typically dictate the fine aerosol mass concentrations (PM_2.5_; mass of particles with diameters smaller than 2.5 µm) as well as the aerosol climate impacts by taking part in cloud formation and by directly affecting the radiation balance.^4–7^ The formation of haze, and aerosol mass in general, is a result of complex interplay between primary particle emissions, new particle formation (NPF) and secondary mass production, all modulated by the prevailing ambient conditions.^8^

Accumulation mode particles can originate either from direct primary emissions of particles in the 100 nm–1 µm size range or from the growth of smaller particles *via* secondary aerosol formation processes, including condensation of various low volatility vapors and the production of particle-phase compounds through heterogeneous reactions on aerosol surfaces. The smaller growing particles can also originate from primary emissions or they can be formed by nucleation/clustering of various combinations of extremely low volatility vapors in NPF events. NPF events can produce large numbers of very small particles in local or regional scales,^9,10^ but their occurrence usually requires low concentrations of pre-existing aerosols, which act as a sink for the potentially-NPF-producing vapors and small clusters.^11–14^ The relative importance of the different aerosol formation pathways is largely dependent on the relative emission rates of primary particles and different chemical compounds, as well as the meteorological conditions.

Beijing and the Eastern China are areas with strong anthropogenic primary particle emissions, main sources being industry, traffic, power production and residential combustion.^15,16^ Despite the strong primary particle emissions, the majority of aerosol mass typically consist of secondary species^17–19^ and NPF events with significant contributions to particle numbers are observed frequently due to high concentrations of sulfuric acid and dimethyl amine.^10,20,21^ Recently, extremely high concentrations of nitric acid have also been proposed as a possible candidate for initiating NPF in urban conditions, especially during colder temperatures.^22^ The precursors for NPF and aerosol growth in Beijing predominantly originate from anthropogenic emission sources.^23–25^ Especially during summer and in low to moderate pollution loadings, a large fraction of aerosol is shown to consist of organics formed in the photo-oxidation of anthropogenic VOCs (volatile organic compounds). During winter and hazier conditions, the inorganic fraction of the aerosol often becomes more significant as the formation of nitrate and sulfate *via* aqueous processing and catalytic reactions gains effectiveness compared to photochemistry.^18,26^ Similar shifts in the secondary aerosol formation pathways can also occur in the diurnal scale as the decreased solar radiation and temperature and increased relative humidity during night-time can promote aqueous formation and condensation of semi-volatile species.^27^

**Fig. 1:**
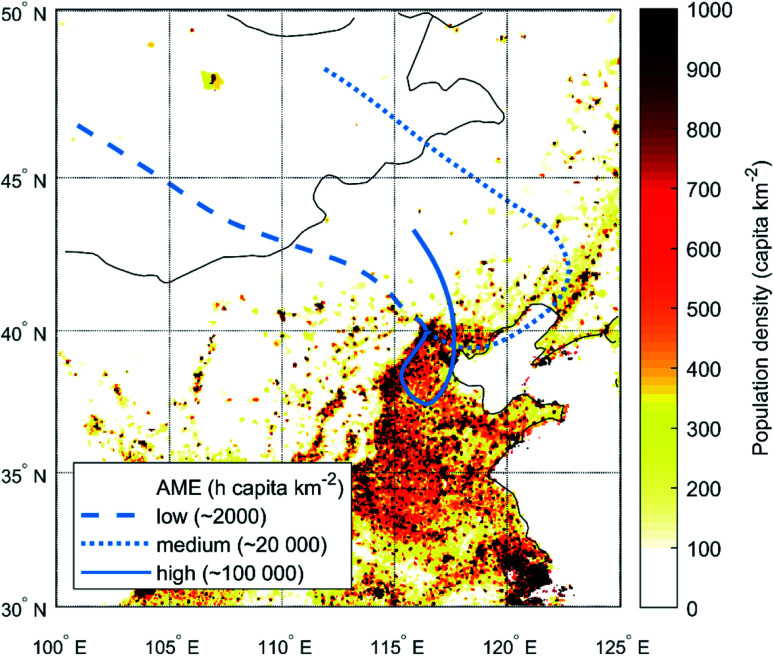
A population density map of north-eastern China displaying example trajectories arriving at Beijing with varying AME_ST,Pop_ values depending on the transport route. Low AME values correspond to northern airflows while high values indicate air masses approaching from the south.

Meteorological conditions can affect aerosol chemical composition and loadings by altering the efficiency and relative strengths of different formation pathways as well as removal mechanisms. However, meteorological conditions can also influence the observed aerosol loadings in terms of changing air mass transport pathways and boundary layer height. Beijing is located on the northern edge of a vast semi-uniform, highly populated area in the north-eastern China (see Fig. 1). Towards the north and the west, the area is confined by mountains. Due to the sparse population and scattered anthropogenic activities in the mountainous area, air masses arriving at Beijing from the north-western sector are typically clean from any major air pollutants. Southern and local air masses, in turn, travel and spend time over the highly populated North China Plain with significant anthropogenic emissions and thus become easily polluted.^28^ The clean north-westerly winds are more typical during winter, when persistent high pressure systems reside over the continental Asia. Connected by the East Asian monsoon cycle, the opposing situation with moist and polluted air masses from the south-east is more typical during summer. However, both southern and northern transport pathways exist during all seasons with significant and widely recognized consequences for the pollution loadings in Beijing.^29^

The variation in pollution loadings caused by the different transport pathways is often further amplified by simultaneous changes in the boundary layer height, which alters the volume of dilution for fresh emissions as well as for the existing concentrations of particles, gases and vapors.^30–33^ The northern transport pathway is typically accompanied with clear sky conditions and higher wind speeds, which promote boundary layer growth compared to the conditions during southern transport.^28^ In addition, the accumulating pollution itself has been shown to play a role in suppressing the boundary layer due to enhanced scattering and warming towards the top of the boundary layer, which creates a deleterious feedback between the increasing pollution and decreasing volume for dilution.^34–37^

Due to the highly adverse health effects of air pollution, significant efforts have been made to understand the complex intricacies of aerosol formation in China.^8,17,19,38–40^ Even so, many aspects of the complete picture require further investigations. One of these aspects is the role of NPF in haze formation. Even though the majority of particle mass during haze has been identified to originate from secondary mass formation rather than direct particle emissions,^17,40–42^ it is much less clear how important secondary particles produced in NPF events are for providing the initial ‘seed aerosol population’ for further growth and mass accumulation compared to primary particle emissions. Some studies suggest that NPF is the main driver of haze formation as haze episodes are consistently preceded by NPF events in Beijing.^19,38,43^ While this chronological connection seems to exist, it does not prove a causality between the two events: in Beijing, NPF events are found to occur in clean northerly air masses which lower the condensation sink,^21,28,44,45^ and since haze events are, by definition, preceded by clean periods, they also become preceded by NPF events.

Understanding the causes of air pollution is vital for its efficient mitigation. However, even when targeted emission reductions are made, evaluating their real life impacts is not trivial due to simultaneous and uncontrollable changes in meteorology that will also affect the observed changes. The meteorological influence is especially important in shorter time scales – an issue that has recently become increasingly clear when estimating the effects of COVID-19-related emission changes on the observed pollution levels.^46^ Several methods exist for estimating the influence of meteorology on air pollution *e.g.* running chemical transport models with constant emissions,^47^ parametrising the effects of meteorological variables using multiple regression analysis^48^ or using machine-learning techniques with meteorological inputs.^49^ While all of the aforementioned approaches are certainly sensible and have their intended uses, machine-learning techniques and regression modelling can be criticized for lacking physical connections, and while chemical transport modelling is likely the most comprehensive method, it naturally relies on the correct description of all the relevant processes and is computationally very expensive.

In this study, we explore and apply a method that combines a core part of chemical transport modelling, in terms of air mass back trajectories and emission inventories, with regression modelling in an effort to estimate the effects of meteorological conditions on aerosol loadings in Beijing in a physically sensible and intuitive way, while retaining simplicity and easy applicability. Our method builds upon the *a priori* knowledge on the importance of anthropogenic emissions for aerosol formation in this atmospheric environment. We calculate the degree in which the air masses arriving at our measurement site in Beijing have been exposed to anthropogenic emissions prior to their arrival, and use this quantity to describe the baseline potential for secondary aerosol formation and primary aerosol accumulation in said air mass. By using fixed anthropogenic emissions, this effectively quantifies the influence of air mass transport, while the effects of other relevant meteorological parameters are investigated and applied *via* residual analysis.

In the first part of our study, we calculate the air mass exposure to anthropogenic emissions (AME) using population density as an estimate for the spatial intensity of anthropogenic emissions, and investigate how new particle formation events and the aerosol size distribution in general behave as a function of this quantity. Our objective is to explore the appearance and temporal development of NPF events as well as the influence of small growing particles from NPF or primary emissions to the development of haze. In the second part, we experiment with different ways of calculating the AME in order to find the most relevant description to be used in the regression model for predicting observed aerosol volume. Finally, we derive a similar model for PM_2.5_ and use it to estimate the changes in aerosol formation efficiency caused by the emission changes during the COVID-19 lockdown period.

## 2 Measurements and methods

### 2.1 Measurement site and instrumentation

The measurements used in this study were performed at the Aerosol and Haze Laboratory at the west campus of Beijing University of Chemical Technology (39.94°N, 116.30°E) between Jan 2018 and Dec 2020. The observations were performed out of a window on the fifth floor of a university building around 20 m above ground level. The location by the 3^rd^ Ring Road is typical of an urban megacity environment, subject to major pollution sources including traffic, cooking and long-range transport of pollution^9,16,50,51^ and frequent NPF events.^19,21,25,52^

The particle number size distribution between 1 nm and 10 µm was measured with two homemade systems, *i.e.*, a diethylene glycol scanning mobility particle sizer (DEG-SMPS)^53,54^ and a particle size distribution (PSD) system^55^ including another SMPS for a larger size range and an aerodynamic particle sizer (APS). The DEG-SMPS covers the size range of 1–6.5 nm and the main parts include a sampling line that minimizes diffusion losses for the smallest particles,^56^ a soft X-ray aerosol neutralizer, a homemade differential mobility analyzer^54^ and a homemade DEG condensation particle counter (CPC). The second SMPS measures particles from 6 to 800 nm and it consists of a soft X-ray aerosol neutralizer, a long DMA (Model 3080, TSI Corp.), a nanoDMA (Model 3085, TSI Corp.) and a CPC (Model 3775, TSI Corp.). The APS (Model 3321, TSI Corp.) measures the aerodynamic particle diameter in the range of 400 nm to 10 µm.

Due to more continuous data coverage, we also utilized the measurements from a separate differential mobility particle sizer (DMPS). The DMPS is a homemade instrument, which samples through a PM_2.5_ filter and consists of a soft X-ray neutralizer, a short Vienna type DMA and a CPC (Model 3772, TSI Corp.). The DMPS covers a size range from 6 to 800 nm by operating the DMA periodically in two flow modes: smaller size range with aerosol-to-sheath flows of 4 and 20 lpm, and larger size range with 1 and 5 l min^−1^.

Meteorological parameters including temperature (*T*), wind speed (WS) and relative humidity (RH) are measured at the rooftop of the university building by an automatic weather station (Model AWS310, Vaisala Inc.). The mixing layer height (MLH) is estimated from the vertical profiles of ceilometer (Model CL51, Vaisala Inc.) backscattering coefficient using a three-step idealized-profile method.^57^

In addition to data from our measurement station, we utilize Ministry of Environmental Protection (MEP) PM_2.5_ data (hourly concentrations) from the nearest national monitoring station (Guanyuan), obtained from the National Urban Air Quality Real-time Publishing Platform (http://106.37.208.233:20035/, last access: 14 October 2021) and hourly precipitation data, obtained from the National Meteorological Information Centre (http://data.cma.cn/, last access: 14 October 2021).

### 2.2 Air mass history

Air mass history was studied by calculating 72 hour air mass back trajectories for each hour of the 3 year study period (2018–2020) using a Lagrangian particle dispersion model FLEXPART version 9.02.^58^ In each of the hourly simulations, 50 000 massless and non-reactive tracer particles are initially distributed evenly between 0 and 100 m above the measurement site and then followed backwards in time for 72 hours. The model output contains (1) potential emission sensitivity (PES) fields (also known as air mass retroplumes), which comprise the combined residence times of the tracer particles above the simulation grid cells and (2) the average positions of the tracer particles, which equate to the traditional single back trajectories. The model output is saved every hour and the simulation grid covers an area from 20–60°N and 95–135°E with a spatial resolution of 0.05°. European Centre for Medium-Range Weather Forecasts (ECMWF) operational forecast with 0.15° horizontal resolution, 137 vertical levels and 1 h temporal resolution was used as the meteorological input for the FLEXPART model.

### 2.3 Air mass exposure to anthropogenic emissions (AME)

We use air mass exposure to anthropogenic emissions (AME) in order to describe the changes in aerosol formation potential with respect to changes in air mass source areas in a semi-quantitative manner. The AME values are calculated both using the single trajectories (ST; eqn (1)) and the potential emission sensitivity fields (PES; eqn (2)). With both approaches, the values are calculated in several ways by varying the field that is used for describing the spatial distribution of anthropogenic emissions/activities and the height limitation of the trajectories included in the calculation. When using single trajectories, the AME at time *t* is obtained by summing up the values of the anthropogenic activity field closest to the hourly trajectory locations during the 72 h backward calculation:1
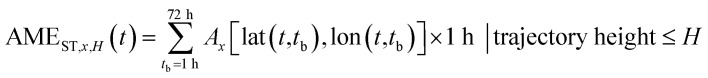
Here ST denotes the use of single trajectories, *x* specifies the field used for describing the level of anthropogenic activities in *A*_*x*_ (see Sect. 2.3.1), *H* is the height limitation for the trajectory (see Sect. 2.3.2) and *t*_b_ is the backward calculation time. The lat(*t*,*t*_b_) and lon(*t*,*t*_b_) stand for the latitude and longitude coordinates of the trajectory released at time *t* after backward calculation time *t*_b_, and *A*_*x*_[lat(*t*,*t*_b_), lon(*t*,*t*_b_)] is the value of *A*_*x*_ closest to the specified coordinates. For simplicity, the trajectory is assumed to have spent the whole hour within the grid cell specified by the momentary hourly location (*i.e.* no interpolation between the hourly output locations is made), which is indicated by the multiplication of each value with 1 h. Limiting the backward calculation time to 72 h essentially acts as a simple weight coefficient for estimating the relevance of emissions towards the aerosol loadings with increasing travel time. In addition to this step-wise decrease in relevance, we briefly tested adding an exponential decay function to continuously describe the potentially diminishing returns from further-away emission sources but found no improvements later on in the analysis. In Fig. 1 we show example single trajectories and the related AME values when using population density as *A*_*x*_ (AME_ST,Pop_). Note that the unit of AME can change depending on the units of *A*_*x*_.

When using the potential emissions sensitivity fields, which directly contain the information of air mass residence times during the past 72 hours, the AME is simply obtained as the sum of the element-wise multiplication between the hourly PES matrices and the anthropogenic activity field:2
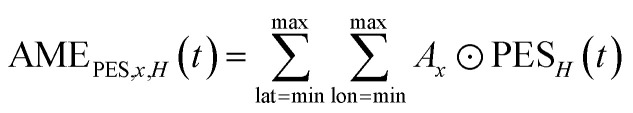
Here the subscript PES denotes the use of potential sensitivity fields, while *x* and *H* again specify the field used for describing the level of anthropogenic activities in *A*_*x*_ and the trajectory height limitation, respectively. For the calculation of AME_PES_, the anthropogenic activity field *A*_*x*_ is interpolated to match the dimensions of the PES field (800 × 800 grid, latitude: 20–60°N, longitude 95–135°E, resolution 0.05°). While computationally more expensive, the emission sensitivity fields should give a more complete description of the air mass movements and source regions.

#### 2.3.1 Fields describing the level of anthropogenic emissions or activities (*A*_*x*_)

The general purpose of *A*_*x*_ in the formulation of AME is to provide a simple estimation of the spatial variability in the overall potential for aerosol formation. Since we know that in Beijing the vast majority of aerosol is of anthropogenic origin, we want *A*_*x*_ to represent anthropogenic emissions. Note that in environments, where other sources are expected to dominate aerosol formation, the *A*_*x*_ field could be changed accordingly, as *e.g.* Tunved, Hansson^59^ use monoterpene emissions to investigate aerosol formation at a boreal forest site. In this study we experiment with four different fields for *A*_*x*_, namely population density (*A*_pop_), SO_2_ emissions (*A*_SO_2__), NO_*x*_ emissions (*A*_NO_*x*__) and NO_2_ tropospheric column density (*A*_NO_2__). The population density data is obtained from Gridded Population of the World (GPWv4.11; CC BY 4,0) and it contains the global population density on the year 2020 with a resolution of 2.5 arc-minutes (∼0.042°). The SO_2_ and NO_*x*_ emissions are from the Multi-resolution Emission Inventory for China (MEICv1.3; http://meicmodel.org, last access 14 December 2021), representing emissions on the year 2017 with a resolution of 0.25° over Mainland China.^60,61^ Lastly, the NO_2_ tropospheric column density is from the OMI/Aura Level-3 daily global gridded (0.25°) nitrogen dioxide product (OMNO2d).^62^

Since our aim with using AME is to describe the effects of changing transport conditions, and not changing emissions, we use time-independent fields for *A*_*x*_. In the cases of SO_2_ and NO_*x*_, we use the annual average emission intensities for the year 2017 and in the case of NO_2_ we use the annual average concentration for the year 2018, which is the first year of our measurements. The fields used in this study are displayed in Fig. 9 in the Appendix.

#### 2.3.2 Use of height limitation (*H*) in the calculation of AME

Anthropogenic emissions mostly occur at ground level and are therefore largely trapped within the boundary layer, particularly in short timescales (<1 week). In the calculation of AME, our aim is to quantify the air mass exposure to these anthropogenic emissions, which is why it is important to consider where this exposure takes place. It is clear that air masses travelling far above the daytime mixing layer will not be affected by the surface emissions, and should therefore not be included in the calculation of the AME. Consequently, one possible way to formulate the height limitation in the calculations would be to only include air masses currently residing within the estimated mixing layer, provided by the ECMWF meteorological data. However, air masses in the residual layer, located above the nighttime mixing layer, are still expected to be exposed to the compounds emitted into the daytime mixing layer,^63^ even if the residual layer is currently uncoupled from the surface. Furthermore, emissions from some high-stack industrial point sources such as power plants may be released above the nighttime mixing layer. Considering these additional interactions as well as uncertainties related to trajectory height and estimations of the mixing layer height, we decided to use constant height limitations around or below the typical daytime mixing layer height. In the case of single trajectories (eqn (1)) the height limitation applies to the average trajectory, while in the case of PES fields, the height limitation is applied to the individual tracer particles. Discussion about the effects of different height limitations and *A*_*x*_ fields in relation to the predictive power of AME is presented in Sect. 3.2.1.

## 3 Results and discussion

### 3.1 New particle formation and the evolution of particle number size distribution as a function of AME_ST,Pop_

Interpreting the connection between NPF and haze can be obscured by the highly varying appearance of the particle number size distributions produced by NPF events in Beijing. On some occasions, the newly formed particles clearly grow from the nucleation mode to Aitken and accumulation mode sizes, where their contribution to the submicron particle mass can become significant,^19^ while in other cases the particles barely seem to grow out of the nucleation mode. Additionally, sudden jumps towards larger particle sizes sometimes occur in the number size distribution, which can make the origin of these larger particles disputable. If the NPF events are clearly observed to produce large particles only under certain conditions, their overall importance towards haze formation remains vague. However, changes in air mass source areas can significantly influence the appearance of NPF event evolution on a fixed measurement site, especially in the case of spatially inhomogeneous surroundings.^64^ Shifts in air masses into areas of stronger emissions can cause jumps towards larger particle sizes, while a spatially limited NPF area could lead to observations of growth stagnation.

**Fig. 2:**
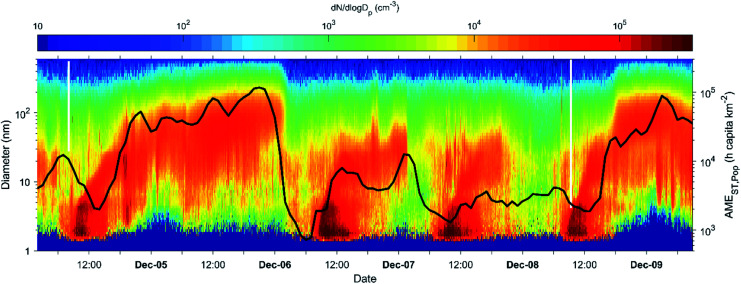
Particle number size distribution (colored fields; left *y*-axis) and AME_ST,Pop_ (black line; right *y*-axis) for a time period during 4–9 December 2018 displaying different kinds of developments after new particle formation events in Beijing, China.

In Fig. 2 we present an example time-series of a particle number size distribution during 4–9^th^ December 2018. On top of the particle number size distribution, we have plotted the 3 hour moving average value of the air mass exposure to anthropogenic emissions using single trajectories, population density and no height limitation in the calculation (AME_ST,Pop_; see Sect. 2.3), to illustrate the connection between the new particle formation events, observed particle diameters and the AME. During the presented five-day period, four NPF events are observed with one NPF event at around noon each day, except on the 5^th^ of December. All of the NPF events occur under clean conditions where the number concentration of particles outside the nucleation mode (particle diameter > 30 nm) is relatively low. During these periods, the loss rate of low volatility vapors onto existing particles is significantly slower, which is likely the main reason enabling the production of new particles *via* nucleation.^21^ These clean periods are clearly represented by low AME values (<10^4^ h capita km^−2^) which result from north-western airflow (see Fig. 10 for air mass source regions during the example period). The daytime situation during the 5^th^ of December clearly deviates from the other days as the AME remains high throughout the day and no NPF event occurs. Regardless of this, an increase in the concentration of roughly 5–30 nm particles is seen around 6 AM, not unlike on the days with NPF events. Based on the size range and the consistent time of appearance, these particles most likely originate from traffic emissions,^16,50^ which also contribute to the nucleation mode particle concentrations in addition to NPF events.

Out of the four NPF events in Fig. 2, only two seem to produce particles that markedly grow past the nucleation mode, while with the other two, on the 6^th^ and 7^th^ of December, the growth is seemingly disrupted as the NPF-related mode disappears from our observations relatively soon after its appearance. In these cases of seemingly disrupted growth, the AME stays low as the source region of the arriving air mass remains in the north-west (Fig. 10). The growth stagnation and the disappearance of the particle mode from our observations suggest that the spatial extent of NPF is limited to nearby areas in the north-west direction.^52,65^ Therefore, a possible explanation for these observations could be that practically no NPF occurs in the clean air masses descending from the mountainous area until they are mixed with precursor vapors emitted in the North China Plain.

In the cases where growth to large particle sizes is observed, also the AME clearly reaches higher values. The increasing AME corresponds to situations where the air mass circulates *via* southern regions before reaching our observation site in Beijing (Fig. 10). This circulation allows for an extended period in the high-emission environment, which seems vital for the production of large particles. In the same manner as increasing AME allows for larger particles, decreasing AME could potentially decrease the size of the observed particles. Some indication of this can be seen during the early hours of December 9^th^. Also, an abrupt decrease in the AME will effectively clean the air from particles as seen around the midnight between the 5^th^ and 6^th^ of December.

It is important to note that both the observed particle size distribution and the AME are dependent on the location of the measurement site. This is especially relevant when considering the NPF events occurring on the 6^th^ and the 7^th^ of December. While the growth of the particles produced in these events is clearly limited in Beijing, the particles will most likely continue growing as they are advected further south within the polluted urban region. This is suggested by the continuous growth with increasing AME after the two other NPF events. Therefore, the appearance of NPF events at a fixed measurement site does not necessarily give a complete representation of their importance towards the formation of larger particles. For example, at a measurement site located in the middle of a vast highly populated region, the AME and the condensation sink could constantly be so high that NPF is very rarely observed, but this would not mean that the high CS at that measurement site could not be contributed by NPF happening upwind. Similarly, the contribution from the growth of other small particles emitted by *e.g.* traffic could be obscured.

**Fig. 3:**
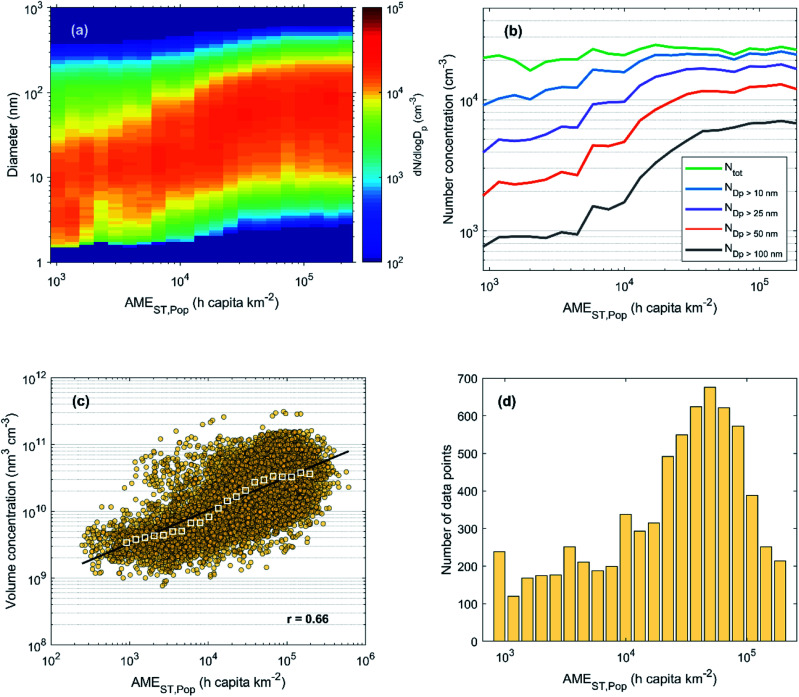
(a) Hourly median particle number size distribution, (b) number concentrations and (c) total volume concentration as a function of AME_ST,Pop_ during 2018–2019 in Beijing. Panel (d) shows the number of data points (hourly median values) in each bin of (a) and (b). For the calculation of the total aerosol volume in panel (c) DMPS data was used instead of the PSD data used in (a) and (b) due to more continuous data coverage and negligible contribution of the smallest (sub 6 nm) particles. In panel (c) the colored circles represent hourly median data and the white squares show the bin median values. The fit and the Pearson's correlation coefficient (*r*) in panel (c) correspond to the hourly (non-binned) data.

While the example period shown in Fig. 2 only gives a short glimpse on the observed connection between particle size distributions and the AME, similar behavior was found throughout the measurement period. In Fig. 3a, we show the median particle size distribution as a function of binned AME values during the years 2018 and 2019. Despite the varying aspects of particle growth events as a function of time (as seen in Fig. 2), the particle size distribution can be seen to represent continuous growth when sorted based on the AME value. This figure can essentially be seen as an illustration of the size distribution development as observed along an initially clean air mass entering and travelling over the highly populated North China Plain region with a continuous supply of anthropogenic emissions for aerosol formation.

In Fig. 3b, we show the median number concentrations of particles in different size ranges with the same *x*-axis as in Fig. 3a. The fact that the number of larger particles (diameter > 50 nm) is low with low AME and increases slowly at first, suggests that these larger particles are neither formed in cleaner air masses nor emitted directly from local primary sources in significant amounts. Instead, these larger haze-forming particles seem to result from the growth of the smaller ones, as the number concentration of the large particles starts to increase much faster after the growing mode reaches these sizes. The close resemblance of the obtained particle number size distribution and that of an NPF event points towards an important role of the growth of nucleation mode (diameter < 30 nm) particles in haze formation. We note, however, that the sub-30 nm particle concentration is affected by both NPF and primary particle emissions from *e.g.* traffic, and that this illustration does not attempt to separate between the importance of these sources. In addition, haze formation could undoubtedly occur also without NPF, as the strong primary particle emissions would most likely be able to provide surfaces for secondary aerosol formation as well as accumulate themselves over time. Yet, the strong influx of particle number and surface area from NPF events is likely to increase the rate at which mass accumulation occurs, therefore affecting the duration, severity and frequency of haze events.^19^

Fig. 3c further illustrates the impact of AME on the observed aerosol loadings by showing the response in total volume concentration. The bin median volumes can be seen to increase nearly linearly (on the log–log scale) with the increasing AME, and even the hourly data shows a moderate to strong positive correlation. With the largest AME values, however, the effect seems to show some saturation. The weakening response to increasing AME is also seen in the development of the number size distribution (panels a and b). This behavior might be an indication of the limited capability of population density in representing the relevant emission for aerosol formation.

### 3.2 AME based regression model for predicting particle volume concentration and PM_2.5_

#### 3.2.1 Comparison of different formulations for calculating AME

Using population density in describing emission from anthropogenic activities provides an intuitive approach to the subject at hand. It clearly highlights the influence of humans on air pollution and displays the harmful combination where the adverse health effects from pollution are amplified due to the large number of people affected. In addition, population density is likely to represent a wide range of emission sources (as well as emissions of different kinds of compounds) *e.g.* traffic, residential cooking and heating, that are all important sources of aerosol. However, emissions from industrial regions are likely underrepresented. In the following sections, we will derive a simple model for estimating particle volume and mass concentration based on meteorological conditions, and for this purpose we want to find if other formulations of AME provide more relevant information in terms of aerosol formation. In addition to population density, we will consider the emissions of SO_2_ and NO_*x*_ from the MEICv1.3 emission inventory^60,61^ due to the known significant contributions of sulfate and nitrate compounds for aerosol in China. We will also consider satellite observations of NO_2_ during the year 2018 for a more purely observation-based description of the spatial variability. The performance of the different approaches is evaluated based on the correlation coefficients between the used approach and the hourly and daily total aerosol volume. The results including the different anthropogenic activity fields as well as height limitations and trajectory types are presented in Table 1.

**Table 1:** Correlation coefficients for total particle volume *vs.* AME using different approaches for the calculation of the AME value (see Sect. 2.3 for more details). Here, the considered trajectory types are single trajectories with either no height limitation (none) or limited to 2 km (a.g.l), and emissions sensitivity fields limited to 100 m, 500 m and no limitation. The considered anthropogenic activity fields are SO_2_ and NO_*x*_ emissions from the MEIC emission inventory, population density and NO_2_ concentration from OMI satellite observation. The correlation coefficients for these 20 different approaches are presented for hourly and daily average values. Overall, the differences between different approaches are small, but using the more informative emission sensitivity fields and estimated emissions of trace gases relevant for aerosol formation provide some improvements

Data	Single trajectories				Potential emission sensitivities					
	Hourly		Daily		Hourly			Daily		
*H*. limit	2 km	None	2 km	None	100 m	500 m	None	100 m	500 m	None
Pop	0.66	0.66	0.72	0.72	0.51	0.63	0.67	0.59	0.69	0.71
SO_2_	0.73	0.73	0.78	0.77	0.76	0.79	0.76	0.81	**0.82**	0.78
NO_*x*_	0.69	0.70	0.76	0.75	0.69	0.76	0.76	0.74	0.79	0.77
NO_2_	0.75	0.76	0.78	0.77	0.71	0.78	0.77	0.81	0.82	0.77

Looking first at the effect of height limitation (see Sect. 2.3.2 for discussion of motivation), we find that with single trajectories limiting the height to 2 km has very little effect. The mean ratio of the AME_ST,Pop_ values with the 2 km limitation to that with no limitation is 0.96 indicating that the trajectories rarely cross the 2 km limit during the 72 h backward calculation time. Mostly similar correlations are also observed using the potential emission sensitivity fields with either 500 m limitation or no limitation, although the mean ratio of the AME_PES,Pop_ values is only 0.78. However, apart from using population density as the activity field, the 500 m limitation generally seems to improve the correlation, indicating some usefulness of the height limitation in describing the relevant surface interactions. While limiting the examination to the lowest 100 m basically ensures that the included air masses are always within the boundary layer, it is clear that relevant information about the air mass movements and surface interactions is lost, as the correlation with the 100 m limit is typically clearly worse than that using the 500 m limit or no limit.

Looking at the case of no height limitation, we can compare the effect of using emission sensitivity fields as opposed to single trajectories. In almost all cases, the correlation is seen to improve with the emission sensitivity fields, as would be expected due to the more informative description of air mass history. However, the improvements are mostly visible with the hourly data, whereas with daily averaging the benefits gained from the more accurate description of momentary air mass source areas seem less significant. Here we would like to remind that in our case, the single trajectories are calculated from the multi-tracer retroplumes, and therefore single trajectories from single-tracer simulations would not necessarily perform equally well.

Comparing the values within each column of Table 1 shows that while no major differences are observed between the different anthropogenic activity fields, using population density always results in the lowest correlation. The population density is likely to describe well emissions from residential and transport-related sources while being less representative of emissions from *e.g.* industrial regions. Thus, on the one hand, the good overall correlation with population density highlights the importance of the former sources while, on the other hand, the better performance of the other fields speaks for the importance of the latter. (Some notable regions with relatively low population density compared to the emissions of SO_2_ and NO_*x*_ seem to be located in Hebei to the east of Beijing and in the coastal Shandong to the southeast of Beijing (see Fig. 9 for maps of the different fields).) The correlations are mostly similar with either NO_*x*_ or SO_2_ emissions, likely due to the shared or co-located emission sources, but SO_2_ seems to perform slightly better. The NO_2_ concentration, which was specifically retrieved for the time-period of this study, often performs similarly to the SO_2_ emissions. It should be noted that in addition to emission intensity, the NO_2_ distribution is affected by atmospheric transport with NO_*x*_ lifetime being up to 1 d.^66^ Overall, the best correlations for both the hourly and daily data are found with using emission sensitivity fields with 500 m height limitation and either the SO_2_ emissions or the NO_2_ concentration. In the following sections we will proceed with using the SO_2_ emissions as the satellite observations are more complicated to retrieve, process and interpret. We note, however, that using satellite data (or population density) provide good alternatives in case emission inventories are not available. In Fig. 11 we have reproduced Fig. 3 but now using the AME_PES,SO_2_,500 m_ instead of the AME_ST,Pop_. Note especially how the saturating effect with high AME values seen in Fig. 3 is no longer observed, again indicating the higher relevance of this metric.

#### 3.2.2 Impact of additional meteorological variables

From the analysis presented this far, it is clear that the air mass transport pathway, and more specifically, the extent of exposure to anthropogenic emissions has a significant impact on the observed particle loadings in Beijing. In the previous section we found that, while the simplest and arguably most intuitive way of describing anthropogenic exposure by using single trajectories and population density performs reasonably well, the ‘more sophisticated’ methods of using potential emission sensitivities combined with emission inventories seem to provide even more relevant information in terms of aerosol formation. Thus, in order to assess the full potential of the method in the development of the regression model, we will continue our analysis with using the AME_PES,SO_2_,500 m_. In the regression model, we will also be using daily averages instead of the hourly data. The intended use of the regression model is to provide a simple way to (1) forecast particle volume and mass concentrations, (2) give an estimate of the impact of meteorology on the observed concentrations *e.g.* to ensure comparability of datasets from different time periods and (3) further evaluate the effect of changes not included in the model *e.g.* changes in emissions to the observed concentrations. For these purposes, the daily averages are often sufficient. Our intent is also to find a description that could be used regardless of the season. When considering the effects of other meteorological variables, the influence of seasonal trends can be difficult to disentangle from the diurnal variability, but using daily averages simplifies the situation. Averaging is also useful for lessening the impact of local pollution plumes which can not be expected to be predicted by our method, but can be a frequent occurrence in a highly urban environment.

In addition to transport conditions, described by the AME, other meteorological factors can contribute to the observed aerosol loadings in several ways. Likely the most influential meteorological factors affecting aerosol formation and the observed loadings are: mixing layer height (MLH), temperature (*T*), relative humidity (RH), precipitation and wind speed (WS). Changes in MLH affect the volume in which the aerosol is distributed in, *T* affects the reaction and condensation rates of different species and RH affects aqueous aerosol formation, which is often discussed to carry significant impact especially to the wintertime aerosol loadings in Beijing. RH can also be an indicator for rainy air masses that could mean increased wet deposition along the transport pathway. Aerosol wet deposition is not included in the calculation of the air mass history and can therefore have significant effects on the relationship between AME and the observed concentrations. However, modelling aerosol wet deposition is also known to be difficult for the models, and its inclusion would not necessarily improve the description. Apart from effects to MLH, wind speed affects the travel times of air masses over different areas and thus the accumulation of pollutants from these areas. However, this effect should already be included in the calculation of the AME, and thus no influence from WS is expected unless other factors are at play. One notable meteorologically influenced factor that will not be examined in this analysis, is the received solar radiation. Solar radiation is important for OH production and thus for the formation of oxidized species that can significantly contribute to secondary aerosol formation. The reason for excluding this effect is that we wanted to use only local and easily accessible measurements. Using local *T* and RH is sensible as they also carry information of advection and are related to a specific air mass, and the local MLH can have strong instantaneous effects to the aerosol loadings, but for radiation one would need to consider the integrated radiation sum along the transport pathway for it to be meaningful. For this, additional observations from several different stations around the actual measurement site would be required, or the received radiation would have to be modelled, which would again have high uncertainties due to difficulties in modelling cloudiness.

**Fig. 4:**
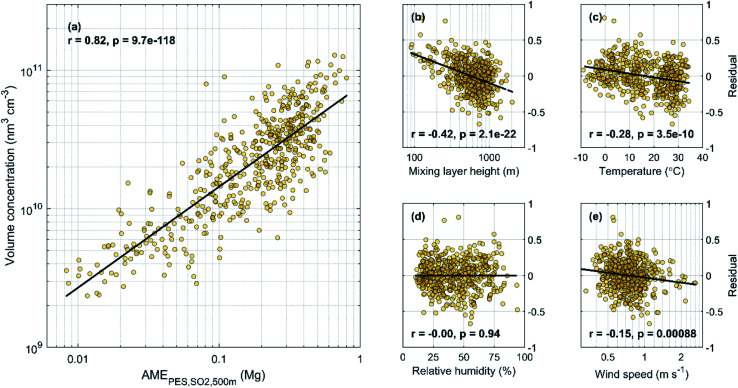
Daily average AME_PES,SO_2_,500 m_*vs.* aerosol volume concentration (a) and the fit residuals (defined as log(*V*)–log(fit)) as a function of MLH (b), *T* (c), RH (d) and WS (e) for the years 2018–2019.

In Fig. 4 we show the daily average aerosol volume concentration as a function of the AME_PES,SO2,500 m_ for the years 2018–2019 and the fit residuals, defined as the logarithm of the ratio between the observed volume concentration and the fit in Fig. 4a, as a function of MLH, *T*, RH and WS. As also shown in Table 1, we find a strong correlation between the AME and the volume (*r* = 0.82), but some dependencies of the fit residuals on the included meteorological variables are also found. The strongest residual dependency is found as a function of the MLH, as the observed values exceed (fall below) the fit prediction at low (high) MLH values, as expected based on the effects of MLH on the volume for dilution. Significant, but clearly weaker negative correlation is found between the residuals and *T*, and a very weak correlation with WS. The correlation with temperature could be related to increased volatility at higher temperatures, but it can also reflect the seasonal changes in anthropogenic emissions, as the emissions are generally lower during summer. A weak positive correlation with the residuals and deseasonalized temperature (*r* = 0.18; not shown) suggest that the latter explanation might be more probable. This is further supported by a very weak positive correlation with the residuals and temperature if the AME is calculated using monthly emissions instead of the annual average. No correlation is found between the residuals and RH.

While practically no dependency on WS is expected, the lack of correlation with RH is more interesting. Many studies discuss the importance of RH for the development of high pollution episodes in Beijing, particularly due to the increased aqueous phase formation of sulfate. A positive correlation between the daily average RH and aerosol volume is also found here (*r* = 0.48; Fig. 12). However, we also find a positive correlation between AME and RH (*r* = 0.59), meaning that the occurrence of high pollution episodes under high RH conditions is at least partly connected to the increased AME values indicating southern air mass transport. Since no RH dependency is found after accounting for the changes in AME (Fig. 4d), some of the effects attributed to elevated RH might actually be related to shifts in air mass transport pathways over generally more polluting areas, as well as to more aged emissions, both reflected by the increasing AME values. The underlying reasons behind the correlation between AME and RH is likely connected to the fact that the air masses arriving from areas of low anthropogenic influence in the north-western sector descend down from the mountains and the associated adiabatic warming will decrease the RH of these air masses, which are likely to be relatively dry already in the first place. Conversely in the south, the subtropical, marine and urban influence can all act to increase RH. Additional links between southern transport and increased RH also exist as, *e.g.* Huang, Ding^31^ shows that southern transport of black carbon from Yangtze River Delta to the upper boundary layer in North China Plain can cause boundary layer stabilisation *via* upper level warming and lower lever cooling, and thus lead to increased surface RH. While, RH is known to alter the formation mechanisms of secondary aerosol,^26^ based on the result shown here, it is possible that the total efficiency of aerosol formation is not strongly affected by the RH conditions. This could be related to *e.g.* stronger photochemical production during low RH conditions. However, this analysis is further complicated by the seasonality of RH (high during summer), whereas the effects of RH on formation pathway are mostly discussed in winter. When looking at the fit residual dependencies separately during winter (Nov–Feb) and summer (May–Aug), still only weak positive correlations are found (*r* ∼ 0.2; not shown). Similar positive correlation is also found when using deseasonalized RH data or AME values calculated using monthly emissions (*r* ∼ 0.2; not shown). While more detailed investigations about the relationships of AME, particle volume and RH are interesting topics for further studies, we will proceed without attempting to isolate the specific dependencies in order to maintain simplicity in the model.

In addition to the meteorological parameters included in Fig. 4, we also investigated the fit residual as function of local daily precipitation. This is done to investigate the effect of wet deposition, which was not included in the trajectory calculations. Somewhat surprisingly, no dependency between the residuals and precipitation was found with the |*r*| and *p* values being ≤0.11 and >0.05, respectively, regardless of the data handling (log or linear scale, days without rain included or not). Similar results have, however been obtained in other studies as well. For example, Sun, Zhao^67^ and Zhao, Sun^68^ investigate the effect of precipitation on the observed PM_2.5_ in Beijing and nearby regions and find varying responses depending on the precipitation intensity and PM loading. Under light precipitation (and especially under low PM), the hourly PM concentrations actually tend to increase, whereas clear PM removal is only observed under intense precipitation (and high PM loadings). Under the typical precipitation (and PM) conditions, the PM concentration seems largely unaffected by precipitation. Since our daily precipitation values do not necessarily represent the varying impacts of different precipitation intensities, we also performed the analysis using daily maximum precipitation values, but the results were essentially unchanged. Therefore, it seems that no simple way of describing the effects of precipitation can be included. We also note, that while removing days with any precipitation from our analysis would improve the correlation coefficients shown in Table 1 by 0.00–0.07, we do not find clear justification for doing so.

**Fig. 5:**
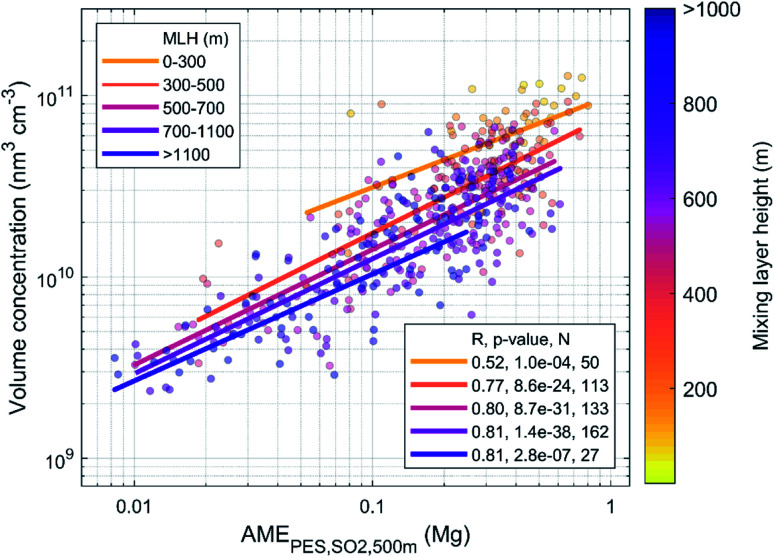
Daily average AME_PES,SO_2_,500 m_*vs.* aerosol volume concentration in different MLH bins during the years 2018–2019.

Based on the results shown in Fig. 4, it seems clear that accounting for changes in the MLH should improve the performance of the predictive model. Since there is a moderate negative correlation between AME and MLH (*r* = −0.42; Fig. 12), we expect the slope of our initial fit to all data in Fig. 4a to be affected by this dependency. In order to quantify the effect of changing AME to volume, without the simultaneous impact from changing MLH, we inspect the dependency with binned MLH data in Fig. 5. We find that the slopes of the fits in different MLH bins stay relatively constant, while the intercept is more clearly affected.

**Fig. 6:**
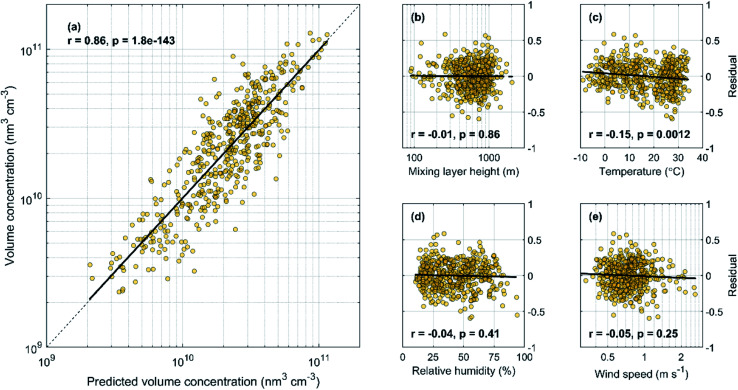
Predicted daily average volume concentration (see eqn (3)) *vs.* the observed volume concentration (a) and the fit residuals (defined as log(*V*_observed_)–log(fit)) as a function of MLH (b), *T* (c), RH (d) and WS (e) for the years 2018–2019.

We then incorporate the MLH dependency into the regression model by using a constant slope, calculated as the observation weighted average of the slopes in the different MLH bins, and by letting the intercept be dependent on MLH. By doing this, we obtain the following equation for the predicted daily average aerosol volume concentration:3
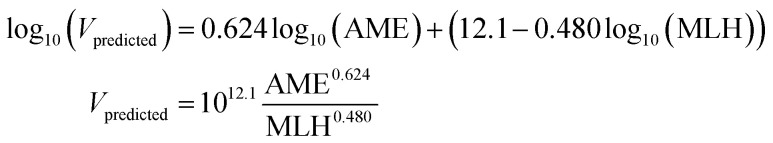
Here *V*_predicted_ has the unit of nm^3^ cm^−3^, AME is the daily average AME_PES,SO2,500 m_ in mega grams of SO_2_ (representing the cumulative SO_2_ mass emitted into the observed air mass during the past 72 h) and MLH is the daily average mixing layer height in meters. In Fig. 6, we show the correlation between the predicted volume concentrations using eqn (3) and the observed volume as well as the fit residuals similar to Fig. 4. We find that accounting for the changes in MLH slightly improves the overall strong correlation (from *r* = 0.82 to *r* = 0.86) and that only a very weak dependency of the fit residual from *T* remains, while RH and now also WS show no correlation. We note that further adding *T* as a predictor only improves the correlation by 0.003 and is therefore not included into the regression model. As such, our final model for predicting aerosol volume concentration only includes the dependencies on AME and MLH and is given by eqn (3).

#### 3.2.3 Applying the model to PM_2.5_ data and investigating the effects of the COVID-lockdown

Despite the grievous consequences of the COVID-19 pandemic, the related lockdown measures have provided significant opportunities for atmospheric scientist to study the effects of abrupt large-scale changes in anthropogenic emissions to air pollution. During the most stringent lockdown measures in China during February 2020, the nationwide emissions of SO_2_, NO_*x*_, non-methane volatile organic compounds (NMVOCs) and primary PM_2.5_ were all estimated to be around 30% lower than during the corresponding period in 2019.^69^ During other months of 2020, the estimated reductions were clearly lower, around 0–10%, for the same components. The emissions from the industrial and transportation sectors were most affected, leading to generally stronger reductions in the highly populated and industrialized parts in the northern and central China.

**Fig. 7:**
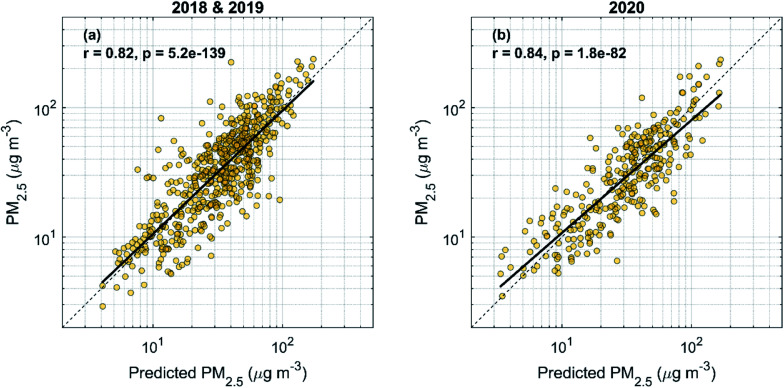
Predicted daily average PM_2.5_ (see eqn (4)) *vs.* observed PM_2.5_ separately for the years 2018–2019 (a) and 2020 (b).

Due to limited data availability at our measurement station during 2020, and because of the larger general interest towards PM concentrations, we use PM_2.5_ data from the nearest national monitoring station (Guanyuan) in this section. Repeating the same steps as in the previous section for volume concentration and using data from the years 2018–2019, we obtain the following equation for predicting the PM_2.5_ concentration as a function of AME and MLH:4
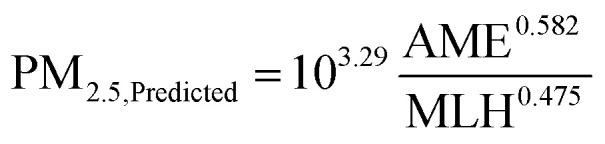
Here PM_2.5,predicted_ is obtained in µg m^−3^ and AME and MLH are the same as in eqn (3). In Fig. 7, we show the resulting correlations between the observed and predicted PM_2.5_ using eqn (4) separately for the model setup period (2018–2019) and for the year 2020. Since no emission changes are considered in the predicted PM_2.5_, the expected result from the reduced emissions in 2020 would be that the observed PM_2.5_ concentrations fall below the predicted ones. However, it is not clear if this is the case, as the fit to the data in Fig. 7b still quite closely matches the 1 : 1 line. Using Student's *t*-test on the logarithm of the ratio between the observed and predicted PM_2.5_, we find that while the data for 2020 does not represent a normal distribution with a mean of zero (with a 5% significance level), the null hypothesis that the data from the 2018–2019 and 2020 periods would be from normal distributions with equal means and variances is only barely rejected (*p* = 0.047). In Table 2, we have listed further statistics on the performance of the predictive model separately for the 2018–2019 and the 2020 periods, as well as for all data combined. In general, the included statistical measures indicate that the predictive model performs quite similarly for the 2020 period but both the normalized mean bias and the mean bias show that the underprediction by the model in 2018–2019 turns into a slight overprediction in 2020. This is in line with the expected behavior where the reduced emissions in 2020 would yield reduced PM_2.5_ concentrations. However, the effect is only on the order of a few µg m^−3^, and the statistical significance of this result is unclear.

**Table 2:** Predictive model performance for 2018–2019, 2020 and all data. FF2: fraction of data points within factor of 2, MB: mean bias (negative values indicate underprediction by the model), NMB: normalized mean bias, RMSE: root-mean-square error and NMRSE: normalized root-mean-square error

	2018–2019	2020	All data
FF2	0.88	0.88	0.88
MB (µg m^−3^)	−3.80	0.68	−2.26
NMB	−0.09	0.02	−0.05
RMSE (µg m^−3^)	22.22	20.69	21.71
NRMSE	0.50	0.53	0.51

**Fig. 8:**
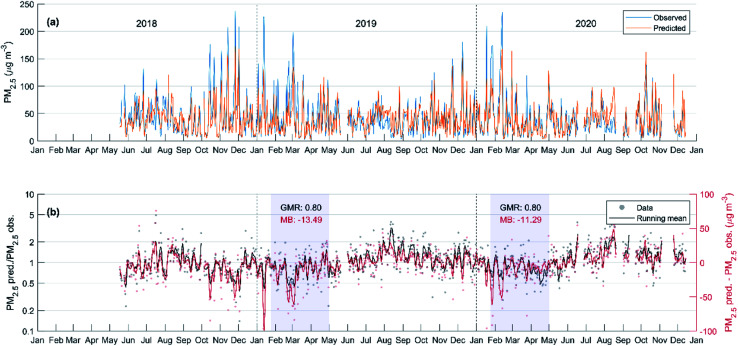
(a) Time series of the predicted and observed daily average PM_2.5_ for the whole period and (b) the relative (left *y*-axis, black) and absolute (right *y*-axis, red) differences between the two. In panel (b) the black and red dots correspond to individual days and the lines display the 7 day moving averages. The highlighted areas in panel (b) show the period of most significant lockdown measures in 2020 and the corresponding period in 2019. The GMR and MB refer to the geometric mean ratio and the mean bias between the predicted and observed PM_2.5_ values during the highlighted time periods.

As mentioned earlier, the most stringent lockdown measures took place in February 2020, and generally the tighter restrictions spanned from late January to April. Therefore, during these periods we would expect to see the clearest differences between observations and the prediction. In Fig. 8a, we show the time series of the predicted and observed daily average PM_2.5_ for the whole study period and in Fig. 8b the absolute and relative differences between the two. The period with tighter restrictions in 2020, and the corresponding period in 2019 are highlighted in grey. Contrary to expectations, the mean bias is quite clearly negative during the tighter restriction, meaning higher-than-predicted PM_2.5_ concentrations. In addition, when comparing the Jan–April period to the previous months, the model seems to be moving from overpredicting to underpredicting, which is also opposite to the expected trend around the onset time of the restrictions. On the other hand, during the corresponding period in 2019, similar behavior and even a larger underprediction by the model is observed, which could suggest other unaccounted factors contributing to the underprediction during this time.

Similar results have also been obtained in other studies. While it is clear that the lockdown caused significant reductions in the emissions of several pollutants, including primary PM, this did not directly translate into similar reductions in the resulting PM concentrations in Beijing. A WRF-Chem study by Huang, Ding^70^ concluded that especially the disproportionate reductions in NO_*x*_ and VOCs enhanced secondary pollution to an extent that could even offset the effect of primary PM reductions. They found that the ratio of the modelled and observed PM_2.5_, without considering lockdown emission changes, did not significantly change as the lockdown measures started, and could also clearly fall below 1 as in our results. Similar findings were obtained by another chemical transport modelling (WRF-CMAQ) study, where the modelled concentrations without including the lockdown effects were on the order of 10–20% larger than the observed ones.^71^ A machine learning study using air mass back trajectories, meteorological parameters and time as predictor variables also found varying influences of the lockdown measures, with the observed values exceeding the predicted ones on average by more than 40% during an early period of the lockdown, while during later stages the observed PM concentrations fell below the predicted ones by more than 30% on average.^49^

When comparing the situation only during the most stringent restrictions in February 2020 with February 2019, we expect the PM_2.5_ in 2020 to be higher by 16.2 µg m^−3^ based on our predictive model. Since the observed increase is only 8.0 µg m^−3^, this would give us an estimate of the PM_2.5_ reduction caused by the reduced emission in Feb 2020 to be around 8 µg m^−3^. These results match well with the results of a GEOS-Chem study by Hammer, van Donkelaar,^46^ where the PM_2.5_ increase due to meteorological conditions in Feb 2020 compared to Feb 2019 is estimated to be around 15–20 µg m^−3^, while the reduction due to emission changes is around 6–7 µg m^−3^.

## 4 Summary and conclusions

In this study, we investigate the evolution of aerosol number size distribution, aerosol volume concentration and PM_2.5_ as a function of air mass exposure to anthropogenic emissions (AME) in Beijing during the years 2018–2020. We define AME as the product of air mass residence time and an anthropogenic activity field during a 72 h backward calculation time. We test the applicability of different anthropogenic activity fields and find SO_2_ emissions to be most suitable considering accessibility and relevance for aerosol formation. We also find benefits from using airmass retroplumes instead of single trajectories and including a height limitation for the considered air masses in the calculation of AME.

Examining the evolution of particle number size distribution in conjunction with AME displays the significant importance of accounting for air mass transport routes when interpreting the appearance and the possible relevance of new particle formation events towards pollution episodes. With limited AME, the NPF events can appear to enter a phase of growth stagnation, even though the formed particles are likely to continue growing outside of our observations. When continuous, cumulatively increasing, input of anthropogenic emissions is provided, we find clear growth with distinct contributions to the number concentrations of large accumulation mode particles. Overall, our results point towards important roles of the nucleation mode particles, formed in NPF events or emitted from primary sources, and secondary aerosol formation in the development of pollution episodes.

We further use the AME as a basis for building a regression model for predicting aerosol volume and mass concentrations with respect to changing meteorological conditions using daily average data during the years 2018 and 2019. When investigating the residuals between the observed concentrations and the initial AME-based prediction as a function of basic meteorological variables (MLH, *T*, RH, WS, precipitation), we find that only MLH provides considerable additional information for the prediction, when considering all data as a bulk. The lack of dependency with precipitation is somewhat surprising but comparable results, suggesting minor changes in PM under typical precipitation conditions, have been found in other studies as well. While the correlation between AME and RH merits further investigations due to possible influence on interpreting the effects of RH on aerosol formation, we decided to omit the examination of more intricate dependencies here to maintain model simplicity. We, however, emphasize the need to account for varying transport conditions when studying aerosol formation in Beijing. The final AME and MLH based regression models for volume concentration and PM_2.5_ both show strong correlation with observations (*r* = 0.86 and *r* = 0.82, respectively), displaying considerable usefulness even with a highly simplified model.

Finally, we apply the PM_2.5_ model for the year 2020 in order to study the effects of the COVID-19 related lockdown measures on PM_2.5_ concentrations. Despite the significant emission reductions, the model was found to match the observations with similar accuracy as during the setup period with only a small change in the overall bias. Focusing on the time period with tighter lockdown measures showed on average higher than predicted PM_2.5_ concentrations, which is contrary to the generally expected behavior from emission reductions. Similar results indicating even increased PM_2.5_ formation during the lockdown around Beijing have, however, been also found by other studies that attribute the finding to increased efficiency in secondary aerosol formation. More generally, these results highlight the complexity of atmospheric aerosol formation and display the necessity of careful planning when targeted emission reductions with the intent of reducing PM_2.5_ pollution are made.

## Author contributions

Conceptualization: SH, MK, PP. Formal Analysis: SH. Funding acquisition: SH, MK, YL. Investigation: JKa, CY, FB, KRD, JJ, YF, CD, LW. Methodology: SH, PP. Project administration: MK, CY, YL, TP, JKu, LD, FB. Software: VV. Supervision: PP, MK. Visualization: SH. Writing – original draft: SH. Writing – review & editing: SH, PP, MK, LD, VV, KRD, FB, JKa, CY, LW, CD, YF, JJ, TP, JKu, YL.

## Note added after first publication

This article replaces the version published on 19th January 2022, which contained errors in eqn (3).

## Conflicts of interest

There are no conflicts of interest to declare.

## Appendix

**Fig. 9:**
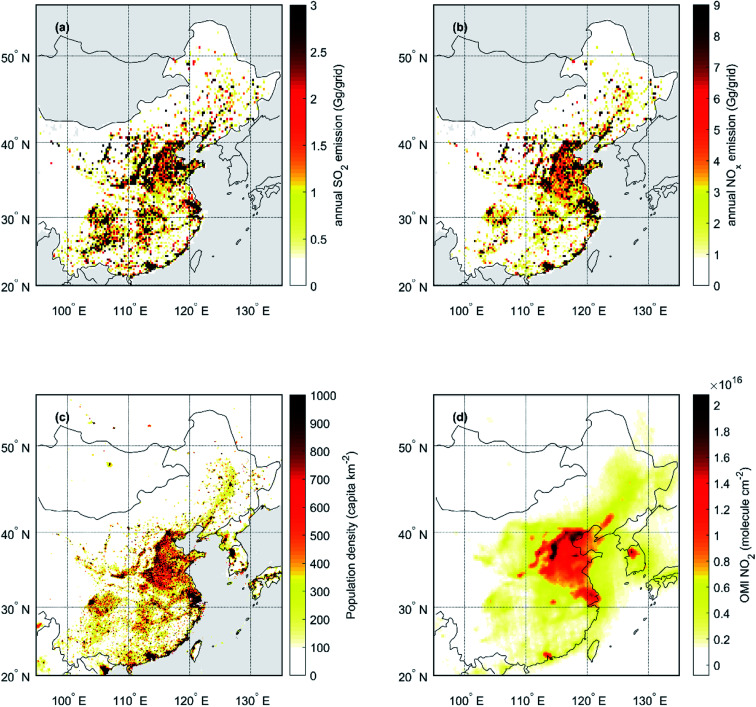
Fields used for describing the spatial variation in anthropogenic activities or emissions (*A*_*x*_). Panels (a) and (b) annual SO_2_ and NO_*x*_ emissions (year 2017) from the MEICv1.3 emission inventory,^60,61^ (c) population density (year 2020) (GPWv4.11; CC BY 4,0) and (d) NO_2_ tropospheric column density (year 2018).^62^

**Fig. 10:**
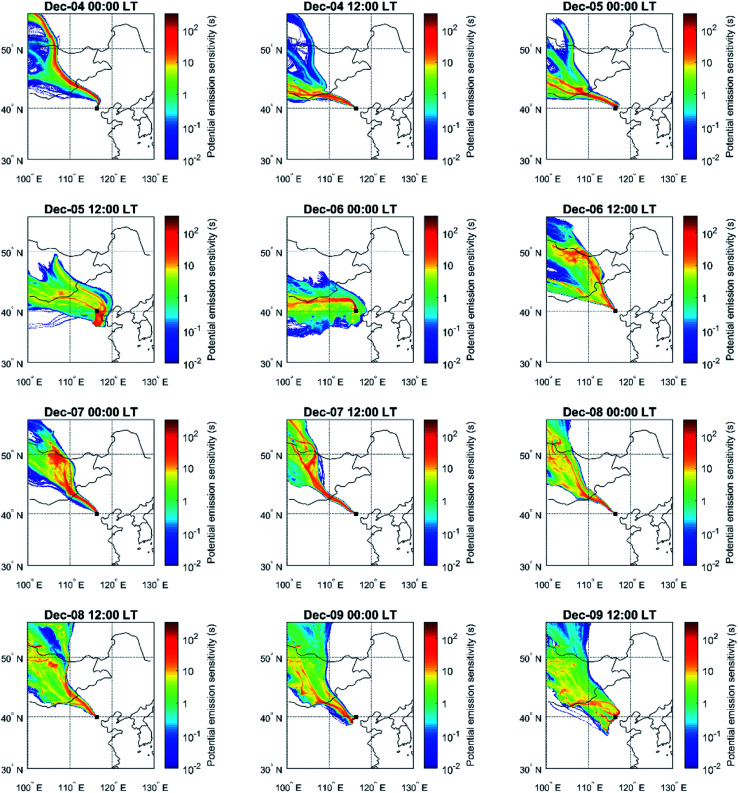
Development of air mass source regions for a time period during 4–9^th^ December 2018 displayed using emission sensitivity fields.

**Fig. 11:**
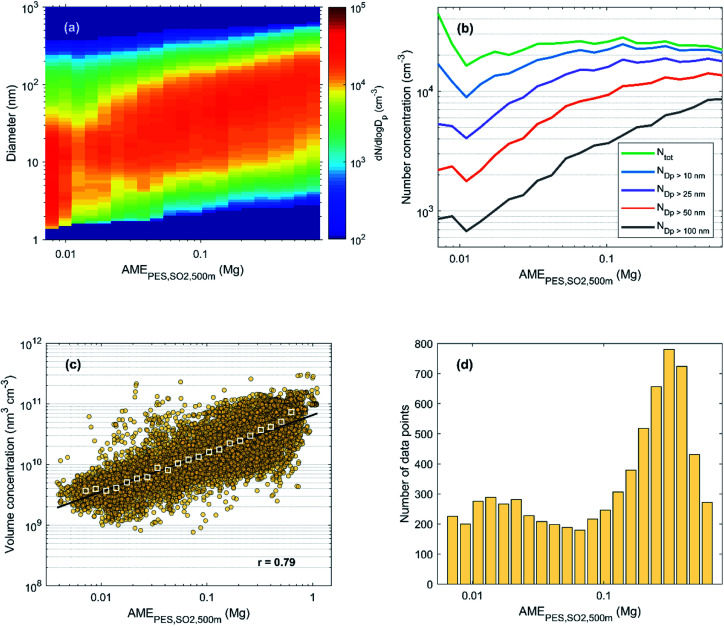
(a) Hourly median particle number size distribution, (b) number concentrations and (c) total volume concentration as a function of AME_PES,SO_2_,500 m_ during 2018–2019 in Beijing. Panel (d) shows the number of data points (hourly median values) in each bin of (a) and (b). For the calculation of the total aerosol volume in panel (c) DMPS data was used instead of the PSD data used in (a) and (b) due to more continuous data coverage and negligible contribution of the smallest (sub 6 nm) particles. In panel (c) the colored circles represent hourly median data and the white squares show the bin median values. The fit and the Pearson's correlation coefficient (*r*) in panel (c) correspond to the hourly (non-binned) data.

**Fig. 12:**
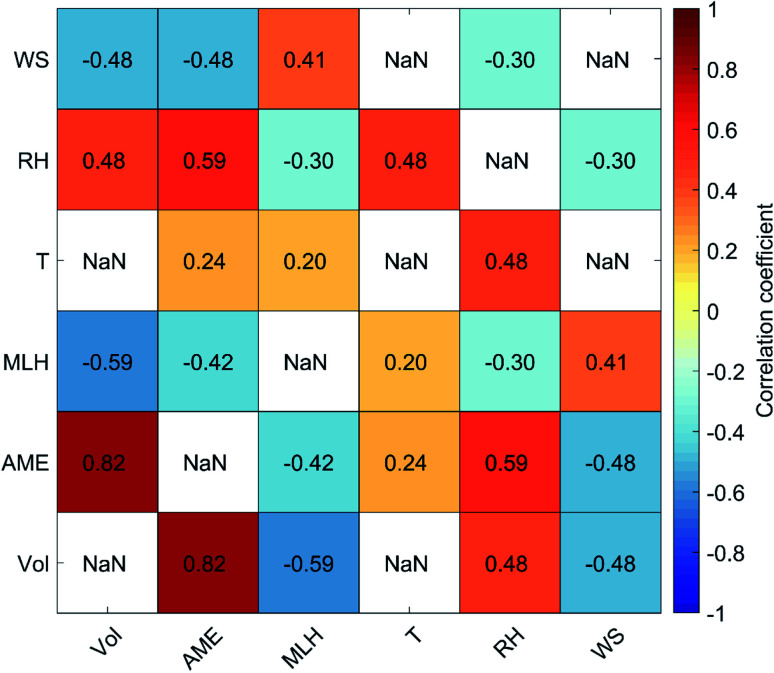
Pearson's correlation coefficients between the daily average values of aerosol volume concentration, AME and different meteorological variables during 2018 and 2019. Logarithmic values are used for all other variables except *T* and RH. Self-correlations and cases where *p* > 0.05 are shown as NaN.

## Supplementary Material

EA-002-D1EA00089F-s001
