# Supplementary material for: Observed coupling between air mass history, secondary growth of nucleation mode particles and aerosol pollution levels in Beijing
Source: Environ Sci Atmos. 2022 Jan 19;2(2):146–64. doi: 10.1039/d1ea00089f (PMC8929417; doi:10.1039/d1ea00089f)
Supplement: EA-002-D1EA00089F-s001 [file EA-002-D1EA00089F-s001.pdf]

Electronic Supplementary Material (ESI) for Environmental Science: Atmospheres.
This journal is © The Royal Society of Chemistry 2022

Appendix A

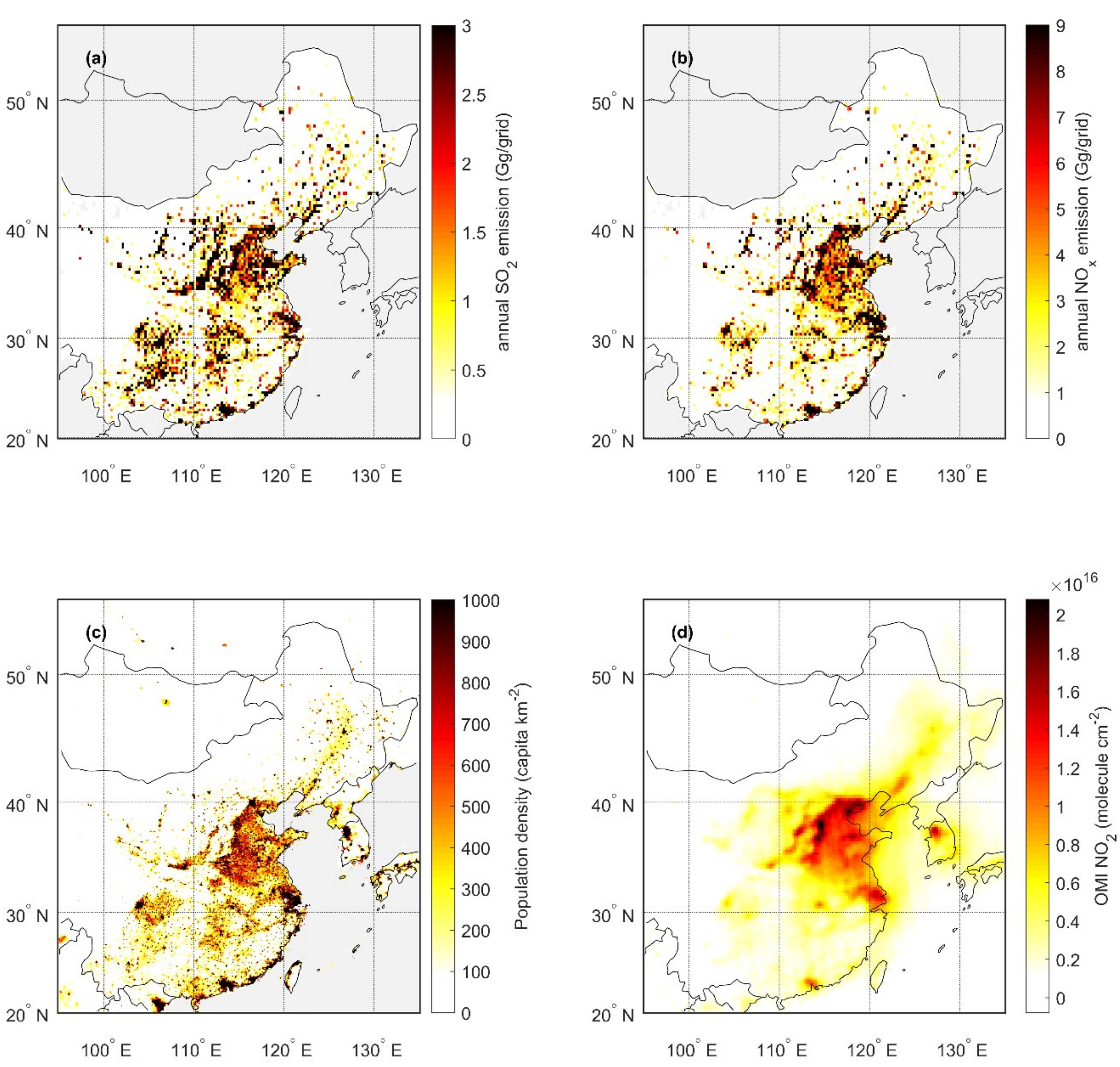

Figure A1: Fields used for describing the spatial variation in anthropogenic activities or emissions (A_x). Panels (a,b) annual SO_2 and NO_x emissions (year 2017) from the MEICv1.3 emission inventory (Li et al., 2017;Zheng et al., 2018), (c) population density (year 2020) (GPWv4.11; CC BY 4,0) and (d) NO_2 tropospheric column density (year 2018) (Krotkov, 2013).

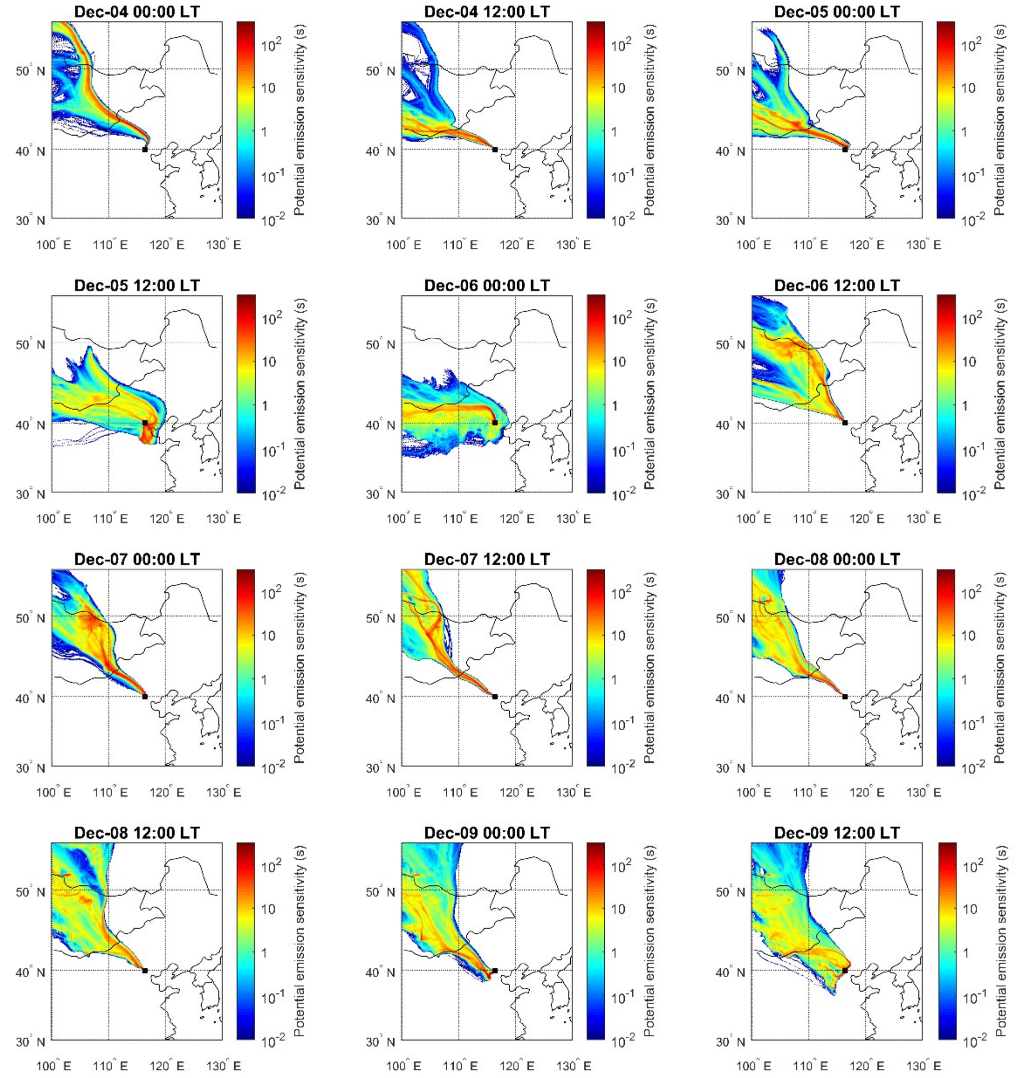

Figure A2: Development of air mass source regions for a time period during 4–9th December 2018 displayed using emission sensitivity fields.

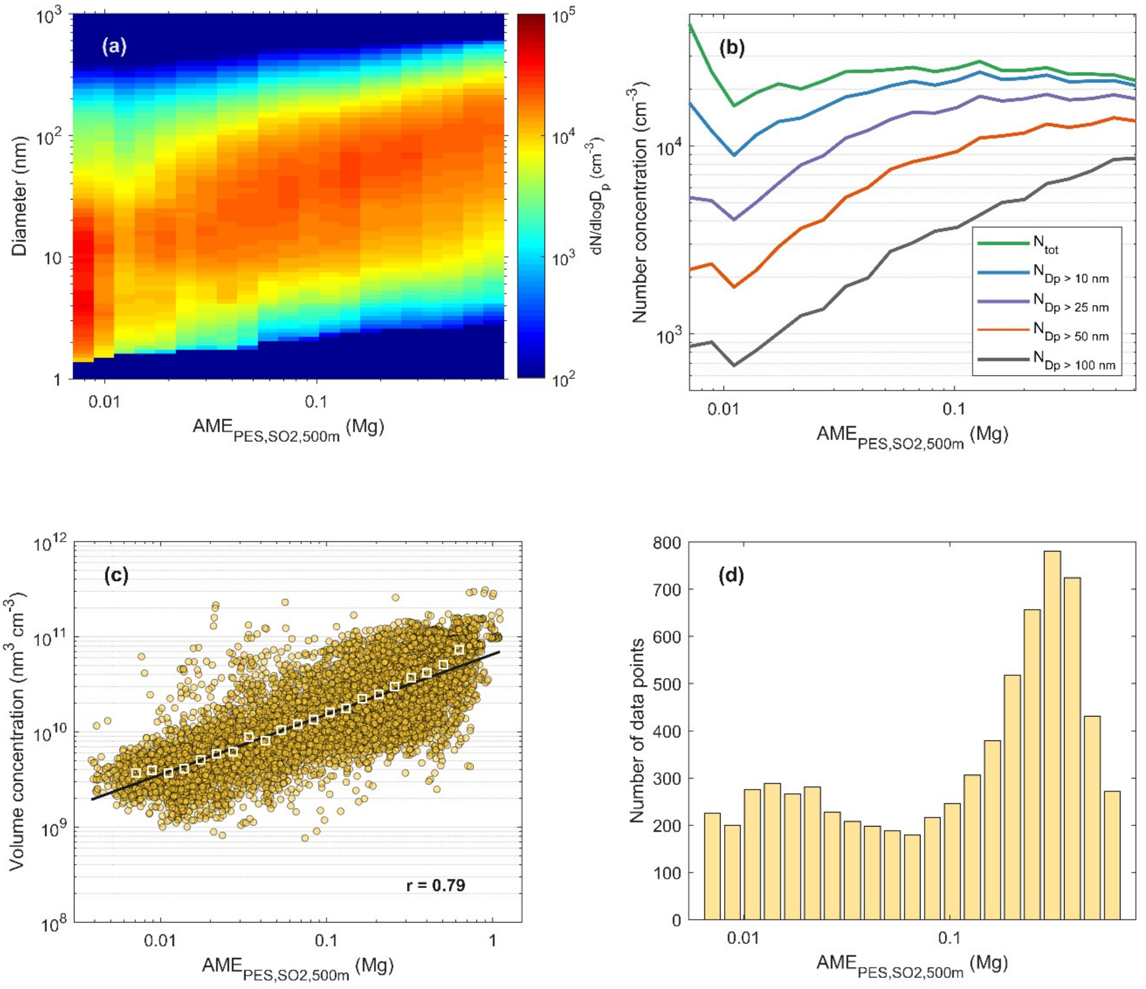

Figure A3: (a) Hourly median particle number size distribution, (b) number concentrations and (c) total volume concentration as a function of $AME_{PES,SO2,500m}$ during 2018–2019 in Beijing. Panel (d) shows the number of data points (hourly median values) in each bin of (a) and (b). For the calculation of the total aerosol volume in panel (c) DMPS data was used instead of the PSD data used in (a) and (b) due to more continuous data coverage and negligible contribution of the smallest (sub 6 nm) particles. In panel (c) the colored circles represent hourly median data and the white squares show the bin median values. The fit and the Pearson's correlation coefficient (r) in panel (c) correspond to the hourly (non-binned) data.

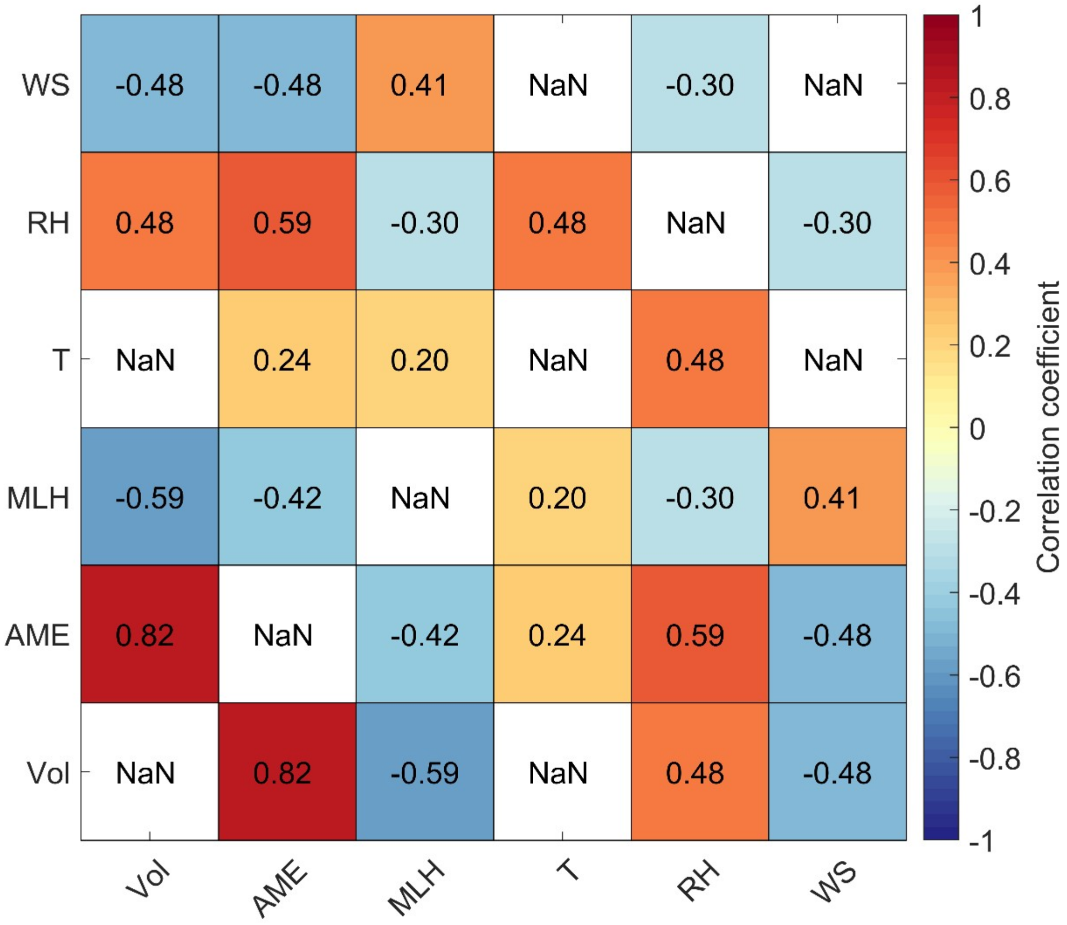

Figure A4. Pearson's correlation coefficients between the daily average values of aerosol volume concentration, AME and different meteorological variables during 2018 and 2019. Logarithmic values are used for all other variables except T and RH. Self-correlations and cases where p > 0.05 are shown as NaN.
